# Editorial: Food rich in phenolic compounds and their potential to fight obesity

**DOI:** 10.3389/fnut.2023.1204981

**Published:** 2023-05-15

**Authors:** Yao Tang, Yuan Huang, Bing Zhang, Ting Luo, Wei Zhong

**Affiliations:** ^1^State Key Laboratory of Food Nutrition and Safety, College of Food Engineering and Biotechnology, Tianjin University of Science and Technology, Tianjin, China; ^2^State Key Laboratory of Food Science and Technology, Nanchang University, Nanchang, China; ^3^Center for Translational Biomedical Research, University of North Carolina at Greensboro, North Carolina Research Campus, Kannapolis, NC, United States

**Keywords:** dietary phenolics, metabolic symptoms, antioxidant, green tea, metabolic pathways

Obesity has become a major public health challenge globally. According to the World Health Organization (WHO), more than half of adults and approximately one in three children in the European Region are overweight or obese (1). While traditional weight loss interventions focus on calorie restriction and exercise, emerging evidence suggests that phenolic-rich foods may hold great potential in fighting against obesity (2).

Phenolic compounds, which are found in many plant-based foods, elicit various protective and preventive effects against obesity, including reducing food intake, decreasing lipogenesis (3), promoting fatty acid beta-oxidation, and reducing inflammatory responses and oxidative stress (4). Recent studies published in the Frontiers in Nutrition explored the potential of phenolic-rich foods in encouraging weight loss and improving metabolic dynamics, making them a promising intervention direction in the prevention and treatment of obesity.

The study by Li et al. examins the potential of phytochemicals as metabolic signals and the mechanisms of these compounds in ameliorating obesity and related metabolic symptoms via regulating specific metabolic pathways. The article highlights the regulation of phytochemicals in toll-like receptor 4 (TLR4), nuclear factor (erythroid-derived 2)-like 2 (Nrf2), peroxisome proliferator-activated receptors (PPARs), fat mass and obesity-associated protein (FTO), and microRNAs (miRNA). Toll-like receptors (TLRs) are a family of pattern-recognition receptors (PRRs) that trigger innate immune and inflammatory responses in response to invading microorganisms and non-microbial endogenous molecules. Phytochemicals with α, β-unsaturated carbonyl groups, including withaferin A, kaempferide, isoliquiritigenin, and curcumin, were found to ameliorate obesity and related metabolic symptoms by suppressing TLR4.

Wang et al. investigated the effects of matcha green tea on gut-liver axis homeostasis in a mouse model of HFD-induced obesity. The study found that matcha green tea ameliorated the development of hepatic steatosis induced by HFD. Dietary matcha supplementation restored fecal bile acid homeostasis and gut microbial symbiosis. Meanwhile, hepatic mRNA expression levels demonstrated that matcha intervention made significant regulatory changes to multiple metabolic pathways of the host, especially glucose, lipid, and bile acid metabolism.

Cao et al. evaluated the effect of solid-state fermentation (SSF) with *Aspergillus niger* on total phenolic content (TPC), total flavonoid content (TFC), individual phenolic contents, and antioxidant and inhibitory activities against metabolic syndrome-associated enzymes in an ethanol extract from *Apocynum venetum* L. The study found that fermentation significantly increased the 2,2'-azino-bis(3-ethylbenzthiazoline-6-sulfonic acid) (ABTS) radical cation, 2,2-Diphenyl-1-picrylhydrazyl (DPPH) radical scavenging, and pancreatic lipase inhibitory activities. TPC showed a significantly positive correlation with antioxidant activities or inhibition against metabolic syndrome-associated enzymes.

Finally, Zhu et al. comprehensively reviewed the polyphenol composition of various sources of propolis, along with the evidence for their anti-obesity effects and their underlying molecular mechanisms. Propolis is a complex resinous mixture produced by honeybees, which contains bioactive compounds. Among these compounds, phenolic compounds have been reported to exhibit various biological and pharmacological effects, and propolis polyphenols have been investigated for their potential as anti-obesity agents in the past few decades. In this article, the impact of propolis polyphenols on obesity-related signal pathways is discussed, and a molecular mechanism of how propolis polyphenols affect these pathways is proposed. The article also summarizes the mechanism by which polyphenols in propolis promote the browning of adipose tissues and their relationship with intestinal microorganisms. The information presented in this article holds great potential for guiding the development of novel drugs to combat obesity and its related metabolic disorders.

These studies emphasize the growing interest in revealing the potential of phenolic-rich foods as a promising dietary intervention against obesity. While more research is needed to fully understand the mechanisms underlying these beneficial effects and to identify optimal doses and sources of these compounds, these findings shed light on the possibility of dietary interventions targeting phenolic-rich foods, which may be effective in preventing and treating obesity and related metabolic disorders. This may involve further research into the specific mechanisms underlying the observed effects, or even clinical trials to determine optimal doses, sources, and delivery methods of these compounds.

Additionally, it is important to consider the broader implications of promoting phenolic-rich foods in the context of obesity prevention and treatment. This may involve examining issues such as food accessibility, cultural dietary preferences, and environmental sustainability, in order to develop reliable interventions that are both effective and equitable.

Overall, the articles discussed in this editorial demonstrate the potential of phenolic-rich foods as a promising dietary intervention in fighting against obesity. As our understanding of these compounds and their biological effects continues to grow, it would be more and more clear that they may offer a valuable addition to existing weight loss and metabolic health interventions and ultimately contribute to the development of more effective and holistic approaches to obesity prevention and treatment.

## Author contributions

All authors listed have made a substantial, direct, and intellectual contribution to the work and approved it for publication.
